# Integrated bioinformatics and machine learning identify key hub genes in early-onset gastric cancer

**DOI:** 10.3389/fonc.2026.1951255

**Published:** 2026-10-14

**Authors:** Shaoxiong Yi, Qiuxia Chen, Jiaxin Wu, Haobin Hou, Huanjie Chen, Weiliang Zhang, Kaiyu Zhu, Baoyi Liu, Peiwen Liang, Shijun Zhang, Dongjie Yang

**Affiliations:** 1 Digestive Medicine Center, the Seventh Affiliated Hospital, Sun Yat-sen University, Shenzhen, China; 2 Guangdong Provincial Key Laboratory of Digestive Cancer Research, Shenzhen, China; 3 Department of Traditional Chinese Medicine, The First Affiliated Hospital, Sun Yat-sen University, Guangzhou, China; 4 Department of General Surgery, The First Affiliated Hospital of Shantou University Medical College, Shantou, China; 5 Center for Gastrointestinal Surgery, the First Affiliated Hospital, Sun Yat-sen University, Guangzhou, China

**Keywords:** bioinformatic, early-onset gastric cancer, hub genes, late-onset gastric cancer, machine learning

## Abstract

**Background:**

Early-onset gastric cancer (EOGC) represents a clinically and biologically distinct subtype of gastric cancer characterized by unique molecular features. However, its underlying molecular mechanisms remain unclear. This study aimed to identify EOGC-associated hub genes using an integrated bioinformatics and machine learning framework.

**Methods:**

Differentially expressed genes (DEGs) between EOGC and late-onset gastric cancer (LOGC) were analyzed using functional enrichment, weighted gene co-expression network analysis (WGCNA), least absolute shrinkage and selection operator (LASSO) regression, and random forest algorithms. Immune infiltration was characterized using CIBERSORT. Candidate hub genes were validated using an independent external cohort, clinical specimens, single-cell transcriptomic datasets, and *in vitro* functional assays. A five-gene logistic regression model was established and evaluated using ROC-AUC, calibration analysis, and bootstrap resampling.

**Results:**

Transcriptomic analysis identified 227 DEGs, and WGCNA identified seven co-expression modules, with the green module showing the strongest association with EOGC. Machine learning identified five hub genes: CCR7, CD1D, PTGER2, PIK3CG, and EBI3. The combined model showed good discriminatory performance in the training, internal validation, and independent external validation cohorts (AUC = 0.909, 0.859, and 0.802, respectively). Bootstrap resampling yielded AUC estimates of 0.908, 0.852, and 0.789, respectively, supporting model stability. All five genes showed higher expression in EOGC and were validated at the mRNA and protein levels. Immune profiling and single-cell analysis revealed an immune-associated molecular phenotype and distinct expression patterns across immune cell subsets. Silencing the hub genes suppressed gastric cancer cell proliferation and migration *in vitro*.

**Conclusion:**

We identified five EOGC-associated hub genes and revealed an immune-associated molecular landscape. The five-gene model represents an EOGC-associated molecular signature distinguishing EOGC from LOGC rather than a stand-alone clinical diagnostic tool. These findings provide insights into the molecular and immune biology of EOGC and may inform future studies of molecular stratification and treatment-related outcomes.

## Introduction

Gastric cancer (GC) remains a major global health challenge, contributing substantially to cancer-related morbidity and mortality worldwide (1). Although the incidence of gastric cancer has declined owing to screening programs and Helicobacter pylori eradication, the incidence of EOGC, defined as GC diagnosed before the age of 45 years, has increased in many countries (2). EOGC represents a biologically distinct subtype compared with LOGC, characterized by a higher prevalence of diffuse-type histology, frequent *CDH1* alterations, a lower frequency of *HER2* amplification, and distinct transcriptional profiles (3). These findings support the concept that EOGC is not merely an early-onset form of LOGC but rather a distinct disease subtype with unique molecular and clinicopathological features. Patients with EOGC often exhibit aggressive clinical characteristics and unfavorable long-term outcomes (3). However, the molecular mechanisms underlying its pathogenesis remain to be further elucidated.

Beyond tumor-intrinsic molecular alterations, the tumor microenvironment, particularly its immune compartment, is increasingly recognized as an important determinant of tumor development, progression, and therapeutic response. Growing evidence suggests that the composition and functional states of immune and stromal populations within the tumor microenvironment play important roles in gastric cancer progression and clinical heterogeneity (4). Nevertheless, the immune landscape specific to EOGC remains poorly characterized. Characterizing the immune landscape of EOGC may provide new insights into its underlying biology and support the development of individualized therapeutic strategies.

The development of high-throughput sequencing technologies has enabled large-scale profiling of cancer transcriptomes, providing unprecedented opportunities to decipher molecular alterations associated with tumor initiation and progression. Integrative bioinformatics approaches, including WGCNA, enable the identification of disease-associated gene modules and biologically relevant molecular networks from complex transcriptomic datasets (5). Moreover, machine learning algorithms, such as LASSO regression and random forest, provide powerful strategies for feature selection and biomarker discovery in high-dimensional genomic data, facilitating the identification of candidate genes and molecular signatures (6).

In this study, we integrated multicohort transcriptomic datasets from public repositories and employed a combined bioinformatics and machine learning framework to identify EOGC-associated hub genes. Furthermore, we characterized immune cell infiltration patterns and explored their relationships with hub gene expression profiles. The candidate hub genes were further validated using clinical specimens, single-cell transcriptomic datasets, and *in vitro* functional assays. This study provides insights into the molecular and immune characteristics of EOGC and highlights potential biomarkers and therapeutic candidates for this distinct gastric cancer subtype.

## Materials and methods

### Data acquisition and preprocessing

Gastric cancer transcriptomic datasets were obtained from the TCGA and GEO databases, including GSE84437, GSE26942, and TCGA-STAD cohorts. Patients were classified as EOGC (<45 years) or LOGC (≥45 years) based on age at diagnosis. Probe IDs from GEO datasets were annotated to official gene symbols, and duplicate probes were averaged. After preprocessing, 1, 018 samples (82 EOGC and 936 LOGC cases) were retained. Batch effects among datasets were corrected using the sva R package (7), followed by log_2_ transformation for subsequent analyses.

### Differential expression and enrichment analysis

DEGs between EOGC and LOGC groups were identified using the limma R package, with DEGs defined by |log_2_FC| > 0.585 and adjusted *P* value < 0.05 (8). The distribution of adjusted *P* values among the identified DEGs can be assessed from the complete DEG results provided in **Supplementary Data Sheet**, which include log_2_FC, and adjusted *P* values. Functional enrichment analysis of the identified DEGs was conducted using GO and KEGG analyses to characterize their biological processes and signaling pathways (9, 10). An adjusted *P* value < 0.05 was considered statistically significant.

### Weighted gene co-expression network analysis

WGCNA was performed using the WGCNA R package to construct gene co-expression networks associated with EOGC (4, 11). The 5, 000 most variable genes were selected for network analysis, and the optimal soft-thresholding power was determined using the pickSoftThreshold function based on scale-free topology criteria. Co-expression modules were identified by dynamic tree cutting, followed by module-trait correlation analysis and assessment of gene significance and module membership to determine EOGC-related modules and candidate hub genes.

### Reactome pathway analysis among different modules

Reactome pathway analysis was conducted using the clusterProfiler R package to characterize the functional relevance of WGCNA-derived modules (12). Module genes were subjected to comparative enrichment analysis using the compareCluster function with enrichPathway as the enrichment method (13). Pathways with adjusted *P* values < 0.05 were considered significantly enriched based on the Reactome database.

### Construction of the training and validation cohorts

The samples were randomly allocated into training and validation cohorts at a 7:3 ratio using the createDataPartition function in the caret R package (14). Stratified sampling was applied to maintain consistent class distributions between cohorts. The training set was used for model development, while the validation set was reserved for subsequent performance evaluation. To further assess the stability of the five-gene model, bootstrap resampling was performed in the training, internal validation, and independent external validation cohorts. Bootstrap-based performance estimates and corresponding 95% confidence intervals were calculated to assess the robustness of the model’s discriminatory performance.

### LASSO regression screening of hub genes

Candidate hub genes associated with EOGC were selected using LASSO regression implemented in the glmnet R package (15, 16). A binomial logistic regression model with L1 regularization was fitted, and the optimal penalty parameter (λ) was selected by ten-fold cross-validation. Genes with non-zero coefficients at λmin were considered candidate hub genes for subsequent analyses (16).

### Random forest model screening of hub genes

A random forest model was developed using the randomForest R package to prioritize EOGC-related candidate hub genes (17). Model performance was optimized based on the out-of-bag (OOB) error rate, with the number of trees evaluated from 1 to 500. The parameter yielding the lowest OOB error was selected for final model construction. Feature importance was subsequently assessed, and genes with importance scores >0.25 were retained for further analysis.

### Diagnostic value of hub genes in EOGC

Receiver operating characteristic (ROC) curve analysis was conducted to assess the discriminative ability of machine learning-derived hub genes for distinguishing EOGC from LOGC. The discriminatory performance of each gene was assessed using the area under the ROC curve (AUC), where higher values reflected greater classification accuracy (18, 19). The robustness of the identified genes was further assessed in the internal validation cohort and an independent external validation cohort using the same approach.

### External validation of the five-gene signature

Two independent GEO datasets, GSE62254 and GSE110875, were used for external validation of the identified five-gene signature. GSE62254 included 28 EOGC and 272 LOGC cases, while GSE110875 included 80 EOGC cases. The two datasets were combined to generate a single independent external validation cohort comprising 108 EOGC and 272 LOGC cases. These datasets were not used for DEG analysis, WGCNA, feature selection, or model construction. The expression levels of *CCR7, CD1D, PTGER2, PIK3CG*, and *EBI3* were compared between EOGC and LOGC cases in the external validation cohort. ROC curve analysis was subsequently performed to evaluate the discriminatory ability of each signature gene, and the corresponding AUC values were calculated.

### Construction and evaluation of the five-gene predictive model

A logistic regression model was constructed based on the expression levels of the five hub genes to evaluate their combined predictive value for EOGC classification. A nomogram was generated using the rms package in R to visualize the contribution of each gene and estimate the probability of EOGC status. The predictive performance of the model was assessed using ROC curve analysis. In addition to the area under the ROC curve (ROC-AUC), sensitivity, specificity, precision-recall AUC (PR-AUC), and balanced accuracy were calculated to provide a comprehensive assessment of model discrimination and classification performance. Corresponding 95% confidence intervals (*CI*s) were also calculated for these performance measures. Model calibration was evaluated using bootstrap resampling with 1, 000 repetitions to compare the predicted probabilities with the observed outcomes. The calibration curve was generated to assess the agreement between model predictions and actual EOGC classification status.

### Identification of immune cell infiltration

The relative abundance of immune cell populations was inferred using CIBERSORT based on transcriptomic profiles (20, 21). The CIBERSORT algorithm was implemented with the CIBERSORT R package using the LM22 signature matrix to estimate the relative proportions of 22 immune cell subsets for each sample. The estimated immune cell fractions were subsequently compared between EOGC and LOGC groups, and the correlations between immune cell populations and the identified hub genes were further evaluated.

### Single-cell RNA sequencing analysis

To determine the cellular distribution of hub genes, a gastric cancer scRNA-seq dataset (GSE150290) was obtained from the GEO database. Single-cell data processing was performed using Seurat (version 5.0.1), including quality control, normalization, identification of highly variable genes, dimensionality reduction, clustering, and UMAP visualization. Cell populations were annotated based on canonical marker genes. Immune cells were further extracted from the integrated dataset and subjected to secondary clustering to refine immune cell subtypes. The expression patterns of hub genes were visualized across immune populations, and a gene signature score was calculated using the AddModuleScore function.

### Experimental validation of candidate hub genes in clinical specimens

Clinical gastric cancer tissues from EOGC and LOGC patients were collected to validate the hub genes identified through bioinformatics analyses. Total RNA was extracted from frozen tissues using TRIzol reagent, followed by reverse transcription and quantitative real-time PCR (qRT-PCR) analysis to determine the mRNA expression levels of *CCR7, CD1D, PTGER2, PIK3CG*, and *EBI3*. GAPDH was used as the endogenous control, and relative mRNA expression was calculated using the 2^-ΔΔCt method.

Expression of the corresponding proteins encoded by the five hub genes was assessed by Western blotting and IHC. Total proteins were extracted using RIPA buffer, separated by SDS-PAGE, and detected using antibodies against the corresponding target proteins. For IHC analysis, FFPE tissue sections underwent antigen retrieval, primary antibody incubation, and DAB-based chromogenic visualization. Protein expression differences between EOGC and LOGC tissues were evaluated using a semiquantitative scoring system based on staining intensity and the proportion of positive cells.

### Functional assays following hub gene knockdown

AGS gastric cancer cells were maintained in RPMI-1640 medium supplemented with 10% FBS at 37 °C under 5% CO_2_ conditions. siRNAs targeting *CCR7, CD1D, PTGER2, PIK3CG*, and *EBI3*, along with a negative control siRNA, were introduced into AGS cells using Lipofectamine™ 3000 (Invitrogen, USA). Cells were subsequently cultured for 24–48 h before functional assays.

### Functional assays

Cell proliferation was measured using the CCK-8 assay according to the manufacturer’s instructions. For colony formation, transfected AGS cells (500 cells/well) were seeded into 6-well plates and cultured for 10–14 days before fixation, crystal violet staining, and colony counting. Cell migration was evaluated by Transwell and wound-healing assays. Briefly, 5 × 10^4^ cells were seeded into the upper chamber of 8-μm pore Transwell inserts with medium containing 10% FBS in the lower chamber. After 24 h, migrated cells were stained and counted. For wound-healing assays, confluent monolayers were scratched with a sterile pipette tip and cultured in serum-free medium, and wound closure was quantified at 0 and 24 h.

### Statistical analysis

All statistical analyses were performed using R software (version 4.2.2). Student’s t-test was used for comparisons between two groups with normally distributed data, and Spearman’s rank correlation analysis was applied to assess relationships between gene expression and immune infiltration. Statistical significance was defined as a two-sided *P* value < 0.05 unless otherwise specified.

## Results

### Transcriptomic profiling reveals immune-associated transcriptional alterations in EOGC

Three independent gastric cancer transcriptomic datasets were integrated for downstream analyses, comprising 82 EOGC and 936 LOGC samples. The overall study workflow is illustrated in **Figure 1**.

**Figure 1:**
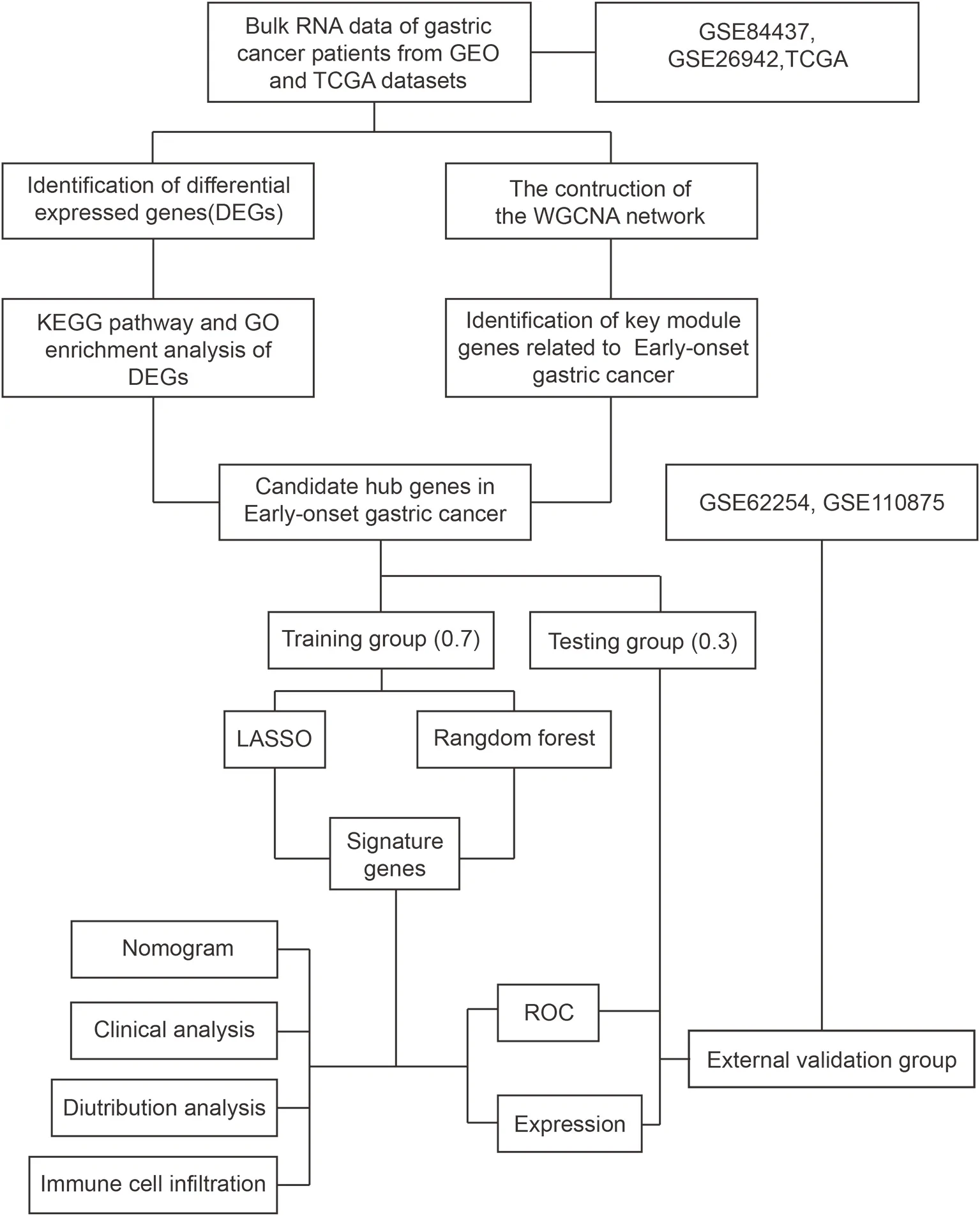
Overview of the study design and analytical workflow.

The clinical characteristics of the integrated discovery cohort were also summarized to assess potential differences between EOGC and LOGC groups. Sex, age, tumor stage, histological subtype, and dataset source were evaluated based on the available clinical annotations and are presented in **Supplementary Table 1**. The EOGC and LOGC groups differed significantly in age and sex distribution, whereas no significant differences were observed in tumor stage or Lauren classification among the clinically annotated samples. Because the completeness of clinical annotation varied among the contributing datasets, not all clinical variables were available for all samples. These characteristics were therefore considered when interpreting the transcriptomic differences between EOGC and LOGC.

Following quality control and batch effect correction, gene expression distributions became highly comparable across datasets, indicating that batch effects were substantially reduced (**Figures 2A, B**). Differential expression analysis identified 128 upregulated genes and 99 downregulated genes in EOGC compared with LOGC, indicating significant transcriptional differences between the two groups (**Figure 2C**). Among the upregulated genes, representative immune-related genes included *CD4, CD1D, PTGER2, CCR7, IL7R*, and *CXCR5*, which are associated with immune cell activation, antigen presentation, and cytokine-mediated signaling. Several gastric lineage-associated genes, including *ATP4A, PGC, PGA3*, and *LIPF*, were also upregulated, suggesting altered regulation of gastric epithelial differentiation-associated programs in EOGC. Conversely, representative downregulated genes included Y-chromosome-related genes such as *RPS4Y1* and *EIF1AY*, as well as *GPA33, HBA2, CA1*, and *NMU*, many of which are associated with metabolic or erythroid-related functions. Given the significant difference in sex distribution between the EOGC and LOGC groups, the differential expression of Y-chromosome-related genes was interpreted cautiously and may partly reflect differences in sex composition rather than EOGC-specific transcriptional alterations. In addition, transcriptional regulators such as *HES6, MSX1, HOXA13*, and *FOXD1* were markedly downregulated, suggesting alterations in developmental and transcriptional regulatory programs. Collectively, these findings indicate that EOGC exhibits a transcriptomic profile characterized by enhanced immune-related gene expression accompanied by downregulation of genes involved in metabolic and developmental processes.

**Figure 2:**
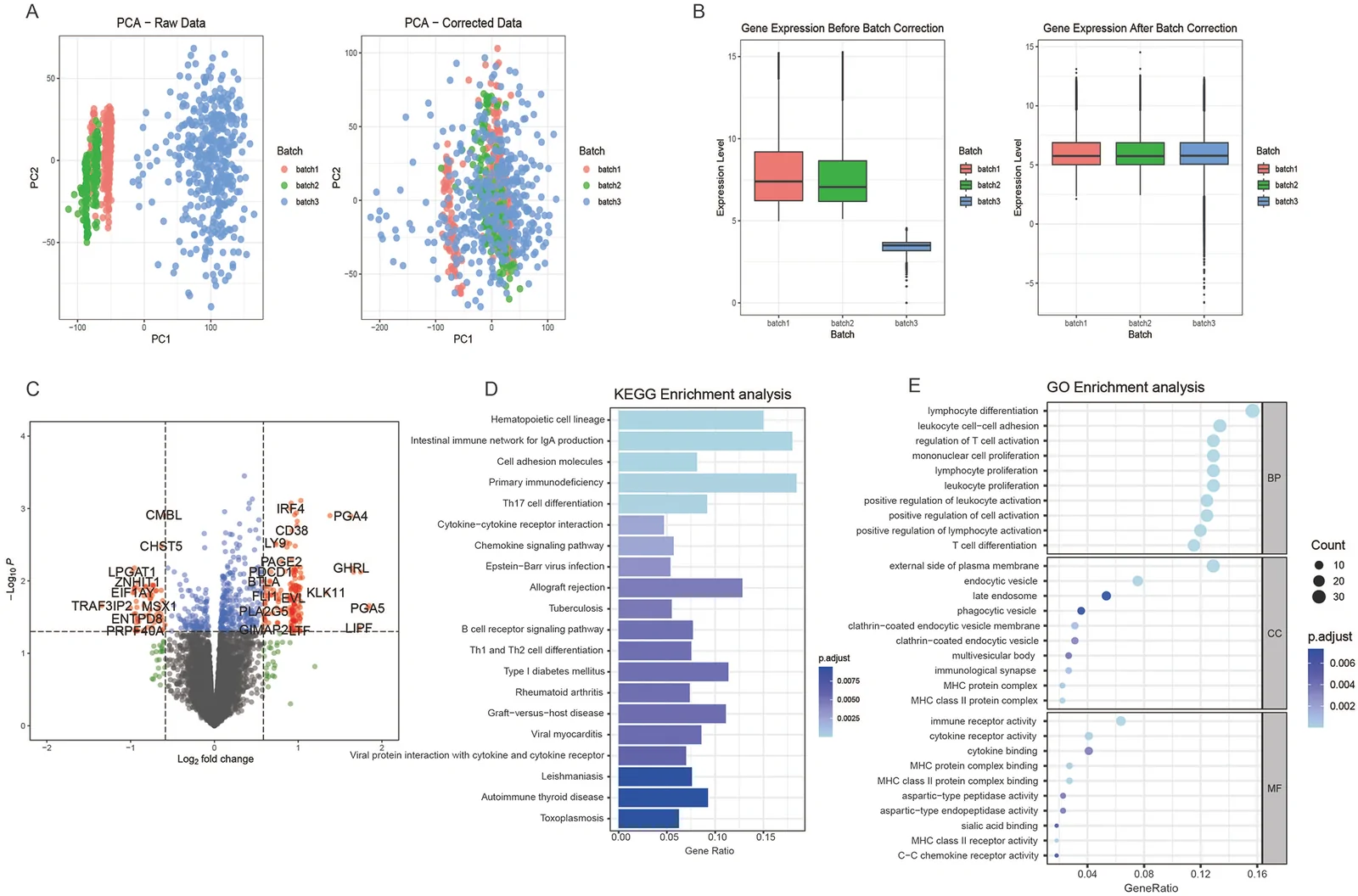
Transcriptomic profiling reveals differential gene expression patterns between EOGC and LOGC. **(A)** Principal component analysis (PCA) illustrating sample distribution before and after batch-effect correction. **(B)** Boxplots showing the comparability of gene expression profiles after batch adjustment. **(C)** Volcano plot displaying differentially expressed genes, with red and blue representing upregulated and downregulated genes, respectively. **(D)** KEGG pathway enrichment analysis of DEGs. **(E)** Gene Ontology (GO) enrichment analysis of DEGs.

Functional enrichment analysis demonstrated that DEGs between EOGC and LOGC were predominantly enriched in immune-associated biological processes. KEGG analysis revealed significant enrichment of immune-related pathways, including B cell receptor signaling, cytokine–cytokine receptor interaction, and chemokine signaling pathways (**Figure 2D**). GO biological process analysis further demonstrated significant enrichment of leukocyte activation, antigen processing and presentation, and interferon-γ-mediated signaling (**Figure 2E**). Together, these findings indicate that immune-related biological processes represent the predominant transcriptional alterations in EOGC, supporting the presence of a distinct immune-associated molecular phenotype.

### Construction of a WGCNA network identifies EOGC-associated gene modules

To characterize EOGC-associated co-expression patterns, WGCNA was applied to the integrated transcriptomic dataset. The optimal soft-thresholding power was selected as β = 9 based on scale-free topology criteria and network connectivity analysis, resulting in the construction of a scale-free co-expression network. Hierarchical clustering subsequently identified seven distinct co-expression modules (**Figures 3A–C**).

**Figure 3:**
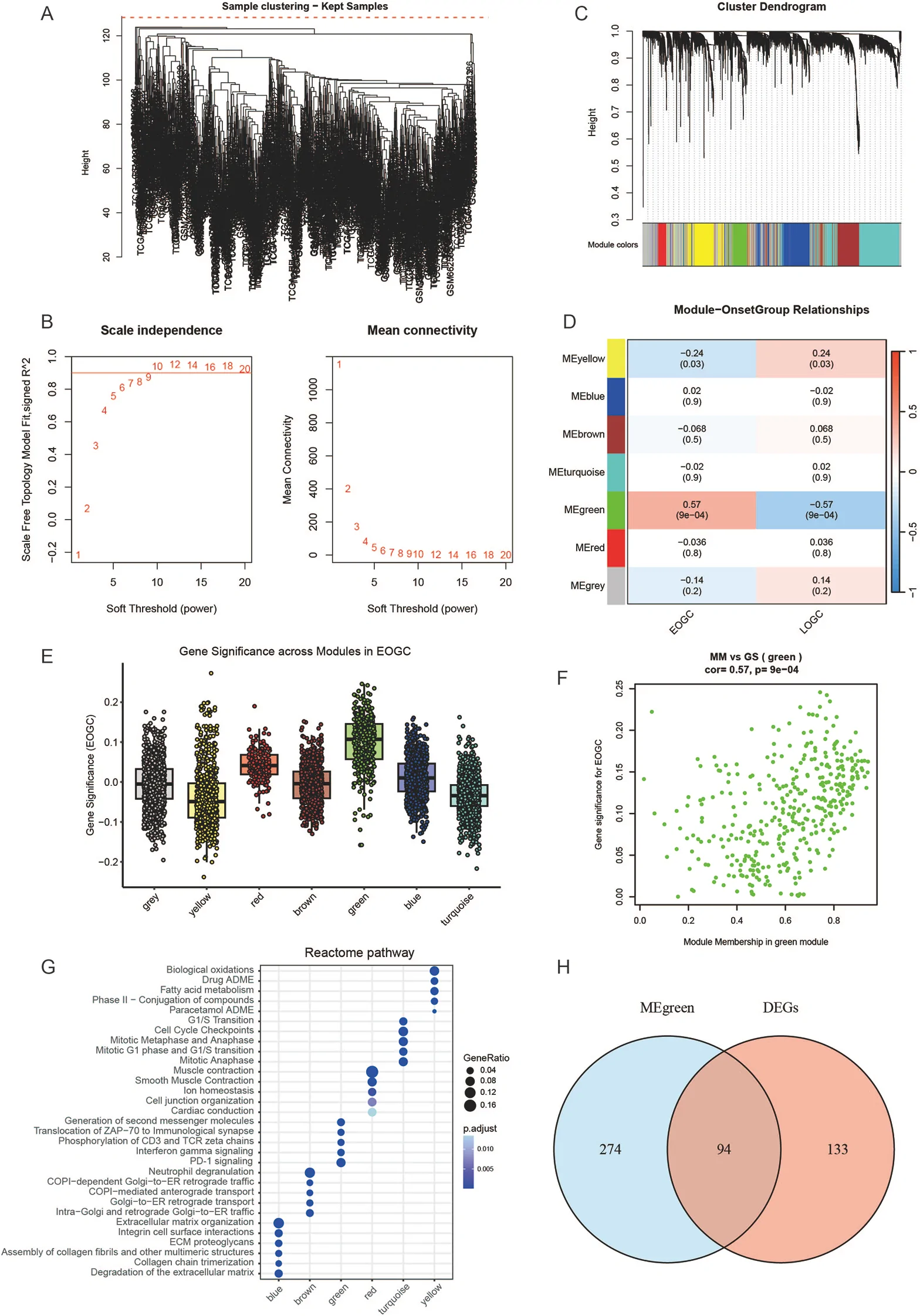
Weighted gene co-expression network analysis identifies EOGC-associated gene modules. **(A)** Sample clustering dendrogram showing sample similarity and detection of potential outliers. **(B)** Selection of the optimal soft-thresholding power based on scale-free topology fit (left) and mean connectivity (right). **(C)** Hierarchical clustering dendrogram of genes based on the topological overlap matrix, with corresponding module assignments. **(D)** Heatmap showing correlations between module eigengenes and clinical traits (EOGC and LOGC). **(E)** Distribution of gene significance (GS) across different co-expression modules in EOGC samples. **(F)** Scatter plot showing the correlation between module membership (MM) and gene significance (GS) in the green module. **(G)** Reactome pathway enrichment analysis of genes from individual co-expression modules. **(H)** Venn diagram showing the overlap between differentially expressed genes and genes in the EOGC-associated green module.

Module-trait correlation analysis revealed that the green module exhibited the strongest positive association with the EOGC phenotype (**Figures 3D–F**). Genes within this module showed high module membership and gene significance values, indicating their strong association with the EOGC phenotype. Pathway enrichment analysis demonstrated that genes in the green module were significantly enriched in immune-related pathways, including B-cell receptor signaling, immune checkpoint-related pathways, and immunoregulatory processes. In contrast, the remaining modules were primarily enriched in extracellular matrix organization, vesicle-mediated transport, cell cycle regulation, and metabolic pathways (**Figure 3G**).

Notably, the green module contained the largest proportion of differentially expressed genes, highlighting its close association with the transcriptional alterations observed in EOGC. Integration of DEGs with genes in the green module identified 94 overlapping candidates, which were subsequently subjected to machine learning-based feature selection (**Figure 3H**).

### Identification and validation of hub genes associated with EOGC

To identify EOGC-associated hub genes, machine learning-based feature selection was performed using the 94 genes overlapping between the EOGC-associated green module and DEGs. LASSO regression identified 10 candidate genes, whereas random forest analysis identified 21 genes based on feature importance scores (**Figures 4A–D**). The intersection of the candidate genes identified by the two machine learning algorithms yielded five hub genes (*CCR7, CD1D, PTGER2, PIK3CG*, and *EBI3*) (**Figure 4E**), which were selected for subsequent validation.

**Figure 4:**
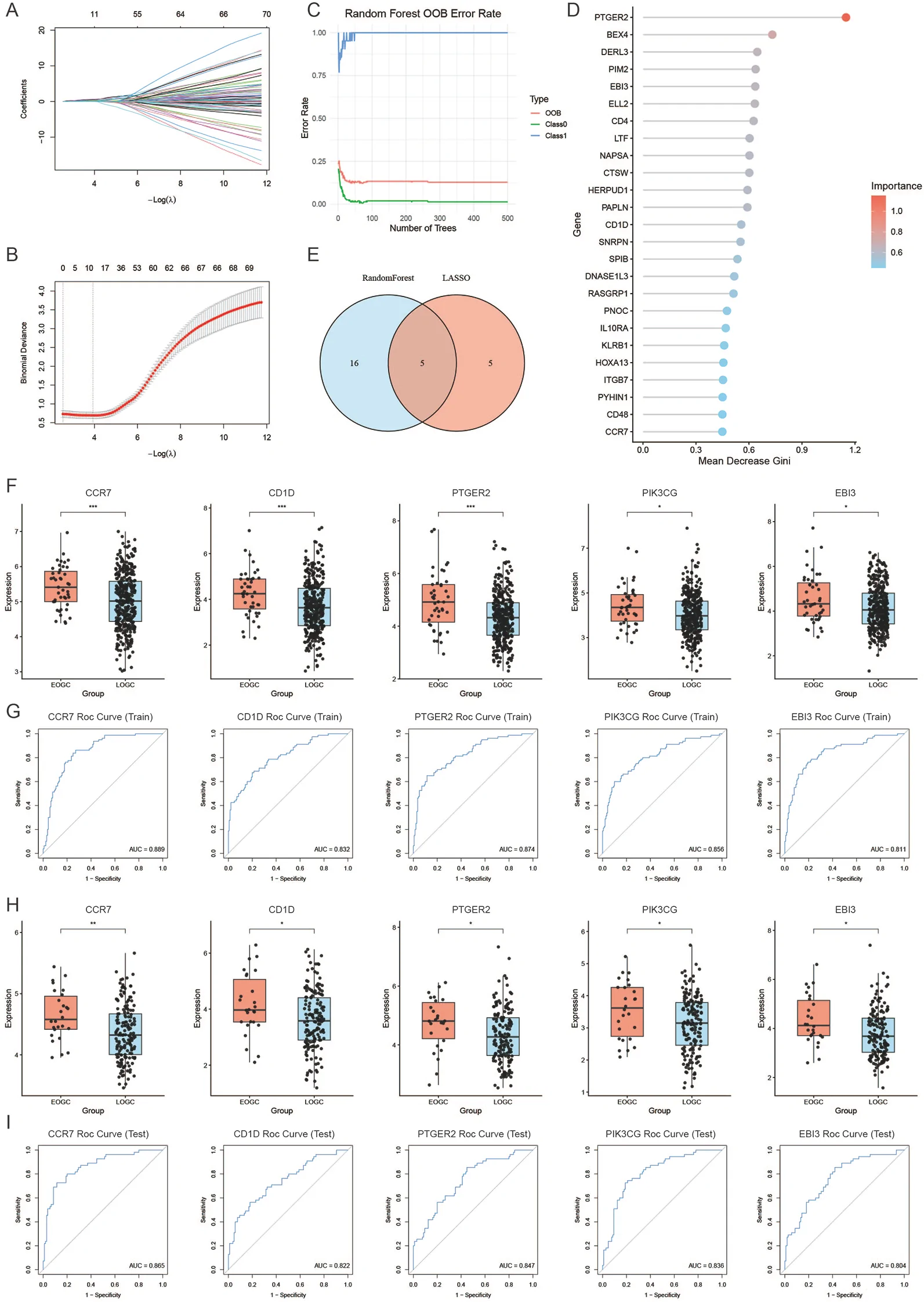
Machine learning-based identification and validation of EOGC-associated hub genes. **(A)** LASSO coefficient profiles of candidate genes in the training cohort. **(B)** Ten-fold cross-validation for selection of the optimal penalty parameter (λ) in LASSO regression. **(C)** Out-of-bag (OOB) error rates of the random forest model across different numbers of trees. **(D)** Lollipop plot displaying the relative importance of candidate genes in the random forest model. **(E)** Venn diagram illustrating the overlap between genes selected by LASSO regression and random forest analysis. **(F)** Differential expression patterns of the five hub genes between EOGC and LOGC samples in the training cohort. **(G)** Receiver operating characteristic (ROC) curves showing the discriminatory performance of the five hub genes in the training cohort. **(H)** Comparison of hub gene expression between EOGC and LOGC samples in the internal validation cohort. **(I)** ROC curve analysis assessing the performance of the five hub genes in the internal validation cohort. * p < 0.05; ** p < 0.01; *** p < 0.001.

In the training cohort, all five hub genes showed significantly higher expression levels in EOGC compared with LOGC (**Figure 4F**). ROC curve analysis demonstrated that all five hub genes showed good discriminatory performance in distinguishing EOGC from LOGC, with AUC values ranging from 0.811 to 0.889 (**Figure 4G**). These findings were further evaluated in the internal validation cohort, where the five hub genes consistently showed higher expression levels in EOGC samples and comparable discriminatory performance, with AUC values ranging from 0.804 to 0.865 (**Figures 4H, I**).

To further assess the robustness and generalizability of the identified hub genes in an independent population, two independent GEO datasets, GSE62254 and GSE110875, were combined to generate a single external validation cohort. The resulting external validation cohort comprised 108 EOGC cases and 272 LOGC cases, and the baseline clinical characteristics of this cohort are summarized in **Supplementary Table 2**. In the independent external validation cohort, all five hub genes were significantly more highly expressed in EOGC than in LOGC samples (**Supplementary Figure 1A**). ROC curve analysis further demonstrated that all five genes showed discriminatory ability for distinguishing EOGC from LOGC. The AUC values were 0.759 for *CCR7*, 0.782 for *CD1D*, 0.800 for *PTGER2*, 0.820 for *PIK3CG*, and 0.771 for *EBI3* (**Supplementary Figure 1B**). These results provided independent evidence supporting the robustness of the five-gene signature in distinguishing EOGC from LOGC.

To further evaluate the combined predictive value of the five hub genes, a logistic regression model incorporating *CCR7, CD1D, PTGER2, PIK3CG*, and *EBI3* was established. The combined five-gene model showed good discriminatory performance in the training, internal validation, and independent external validation cohorts, with ROC-AUC values of 0.909 (95% *CI*, 0.880-0.938), 0.859 (95% *CI*, 0.818-0.899), and 0.802 (95% *CI*, 0.758-0.846), respectively (**Figures 5A–C**). Bootstrap resampling further demonstrated the stability of the model, yielding AUC estimates of 0.908 (95% *CI*, 0.876-0.921), 0.852 (95% *CI*, 0.800-0.878), and 0.789 (95% *CI*, 0.743-0.838) in the training, internal validation, and external validation cohorts, respectively. The close agreement between the original and bootstrap AUC estimates supported the robustness of the model’s discriminatory performance. The corresponding sensitivity, specificity, PR-AUC, and balanced accuracy values are summarized in **Supplementary Table 3**. Calibration analysis showed good agreement between the predicted and observed probabilities, indicating good calibration performance of the model (**Figures 5D–F**). The calibration *P* values were 0.24, 0.18, and 0.33 for the training, internal validation, and independent external validation cohorts, respectively. A five-gene-based nomogram was constructed to estimate the probability of EOGC classification (**Figure 5G**). The nomogram demonstrated that each hub gene contributed to the prediction of EOGC status, with higher total scores corresponding to an increased probability of EOGC. Collectively, these results suggest that the five-gene-based predictive model may serve as a potential molecular classifier for distinguishing EOGC from LOGC.

**Figure 5:**
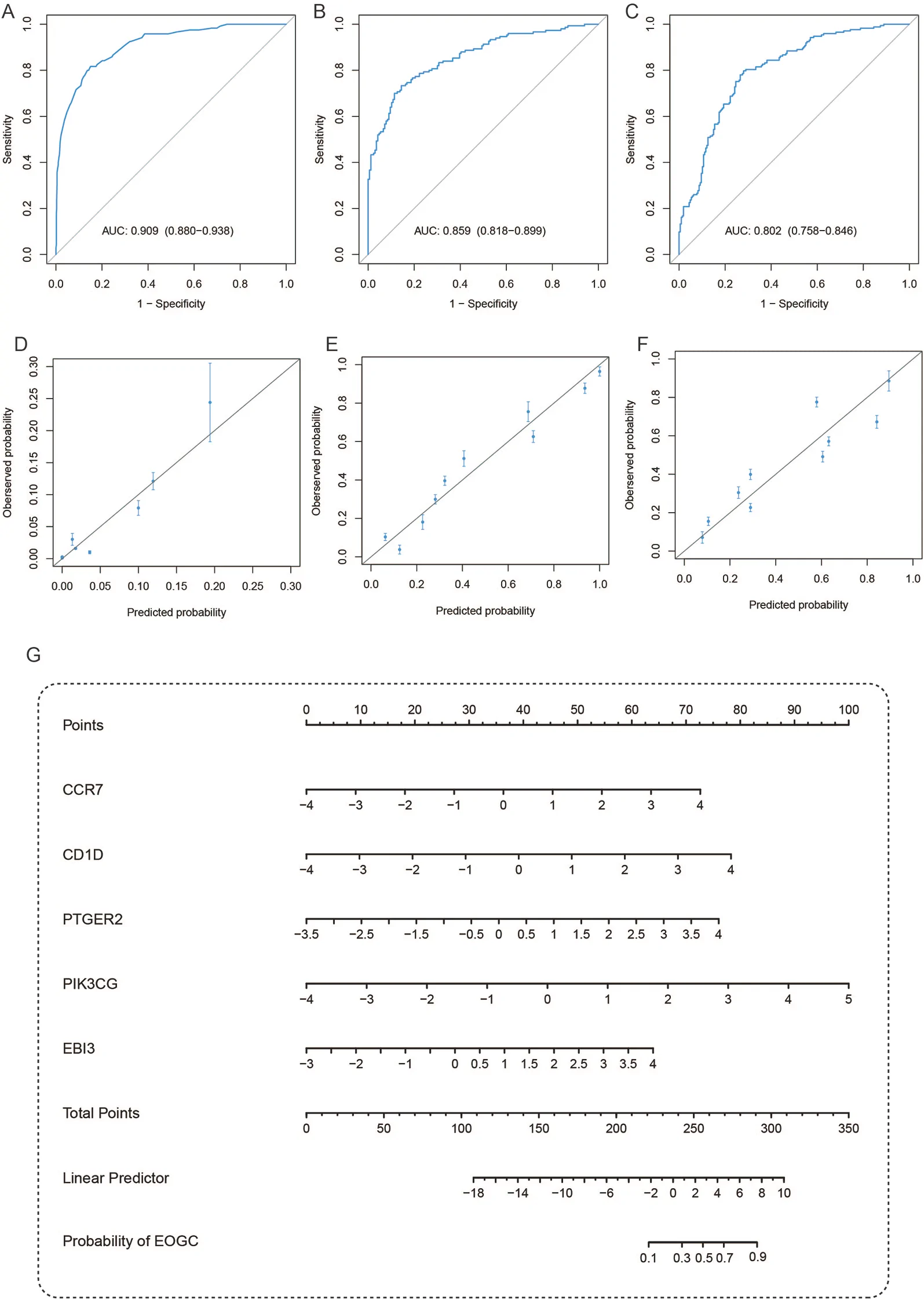
Performance and calibration of the five-gene predictive model. **(A)** ROC curve of the combined five-gene model in the training cohort. **(B)** ROC curve of the combined five-gene model in the internal validation cohort. **(C)** ROC curve of the combined five-gene model in the independent external validation cohort. **(D)** Calibration curve of the five-gene model in the training cohort. **(E)** Calibration curve of the five-gene model in the internal validation cohort. **(F)** Calibration curve of the five-gene model in the independent external validation cohort. **(G)** Nomogram based on the five-gene logistic regression model.

### Immune infiltration landscape and hub gene associations in EOGC

To characterize the immune infiltration landscape of EOGC, CIBERSORT was performed to estimate the relative proportions of immune cell subsets in EOGC and LOGC samples. Significant differences in the inferred abundance of several immune cell populations were observed between the two groups. Compared with LOGC, EOGC samples exhibited increased proportions of naive B cells, activated immune cell subsets, effector T-cell populations, mast cells, neutrophils, eosinophils, and Th1 cells (**Figure 6A**). These findings indicate that EOGC and LOGC possess distinct immune infiltration patterns.

**Figure 6:**
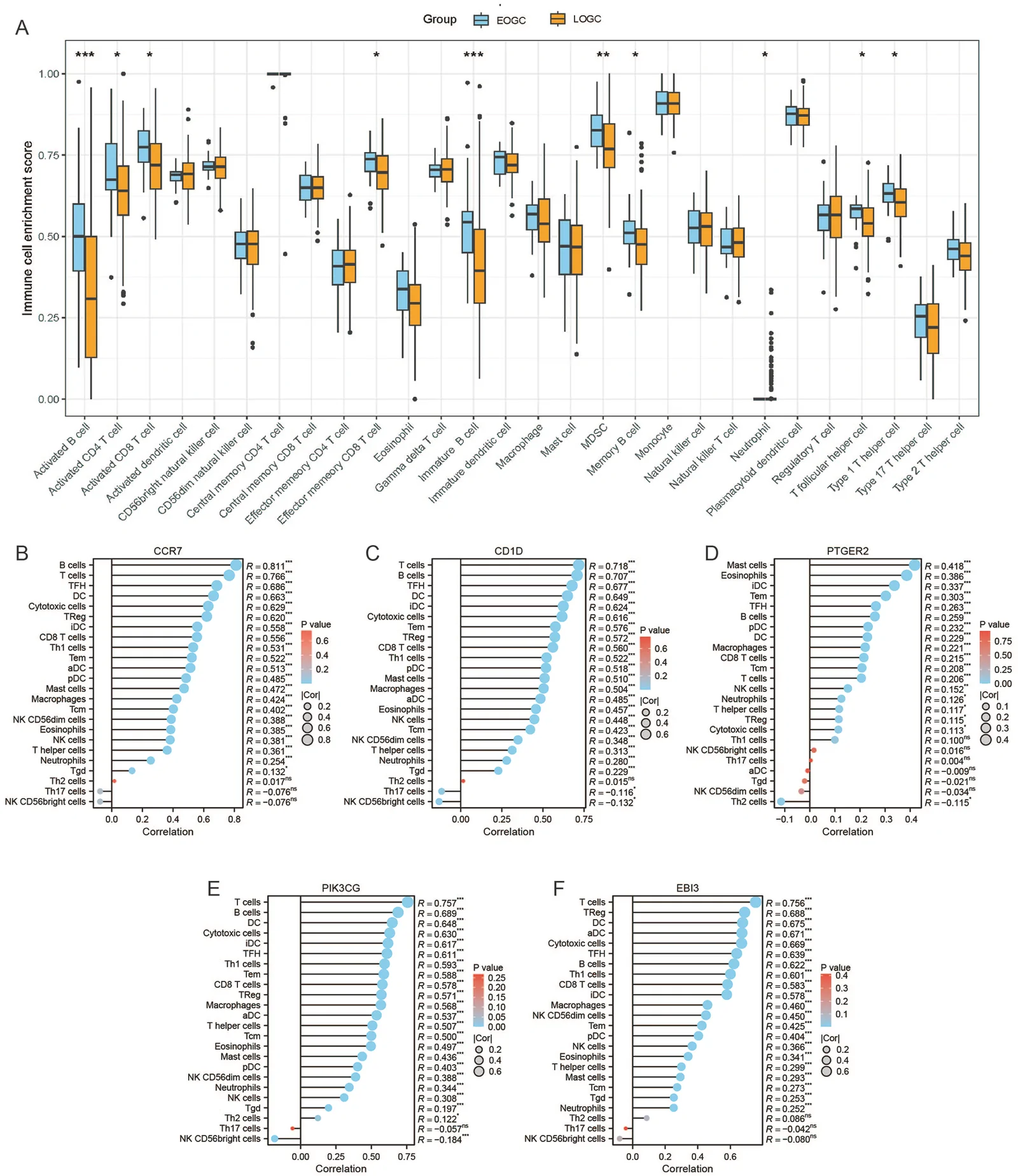
Immune infiltration landscape and association of hub genes with immune cell populations in EOGC. **(A)** Comparison of immune cell enrichment profiles between EOGC and LOGC samples based on CIBERSORT analysis. **(B–F)** Spearman correlation analyses showing the relationships between hub gene expression (*CCR7, CD1D, PTGER2, PIK3CG*, and *EBI3*) and immune cell enrichment scores in EOGC samples. Statistical significance was defined as follows: *P < 0.05; **P < 0.01; ***P < 0.001.

To investigate the potential association between hub gene expression and immune characteristics, Spearman correlation analysis was conducted within the EOGC cohort (**Figures 6B–F**). *CD1D, CCR7, EBI3*, and *PIK3CG* exhibited positive correlations with multiple immune cell populations, including B cells, dendritic cells, and cytotoxic T-cell subsets (r = 0.56-0.81, P < 0.001). *PTGER2* also showed significant associations with several immune populations, although the correlation strengths were relatively weaker. Among these genes, *CCR7* and *CD1D* demonstrated relatively stronger associations with B-cell and dendritic-cell signatures, whereas *EBI3* and *PIK3CG* showed broader relationships with immune-related cell populations. Collectively, these results suggest that the identified hub genes are closely associated with immune infiltration characteristics in EOGC and may serve as potential immune-related biomarkers.

### Single-cell analysis reveals cell-type–specific localization of hub genes in the immune microenvironment

To further characterize the cellular distribution of hub genes, gastric cancer scRNA-seq data were analyzed. Fourteen immune cell clusters were identified, with B cells and T cells representing the predominant immune populations (**Figure 7A**).

**Figure 7:**
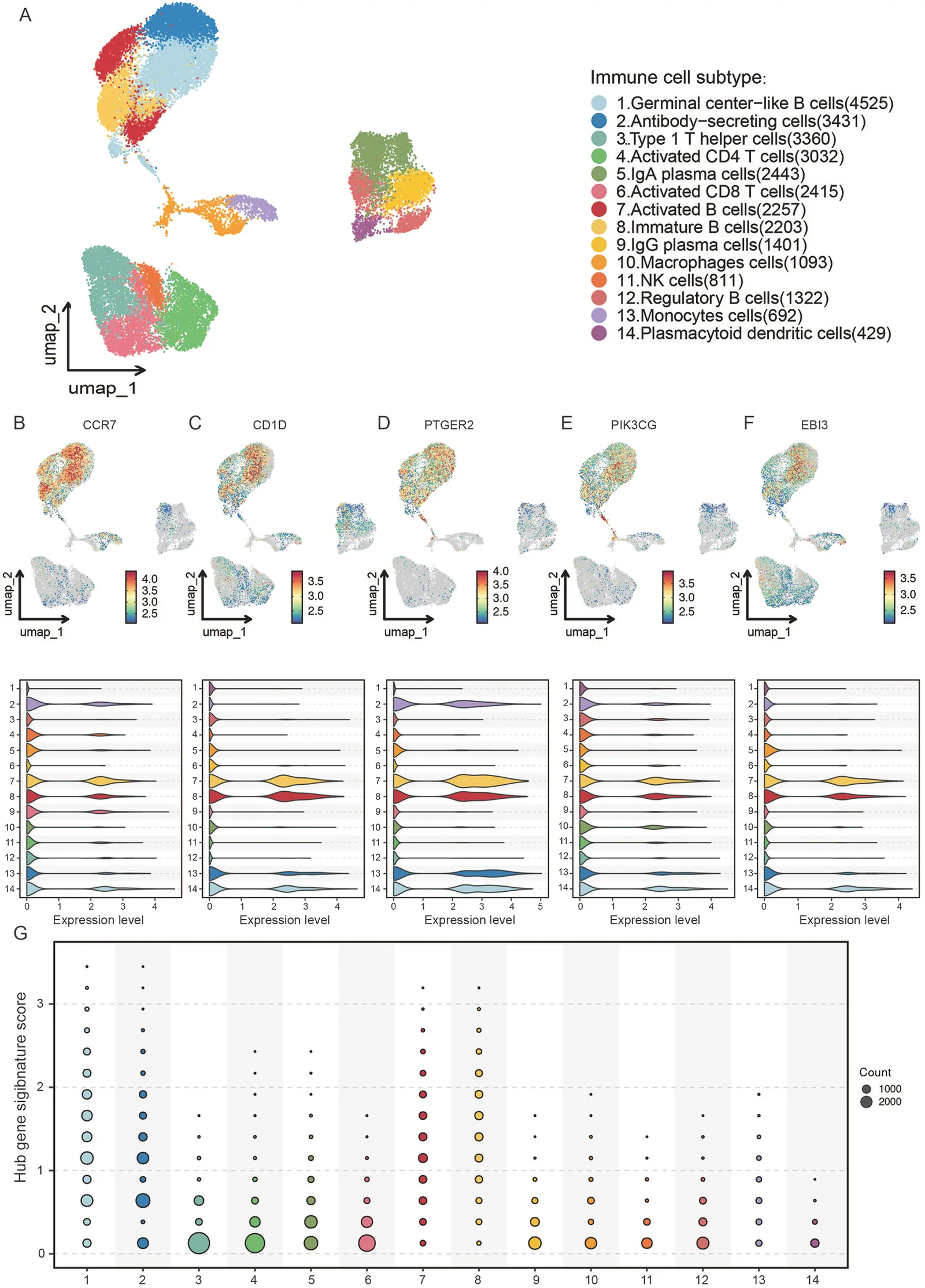
Single-cell transcriptomic analysis defines the cell-type-specific expression patterns of EOGC-associated hub genes. **(A)** t-SNE visualization showing the distribution of 14 immune cell clusters identified from gastric cancer single-cell RNA sequencing data. **(B–F)** Feature plots and dot plots depicting the expression patterns of *CCR7, CD1D, PTGER2, PIK3CG*, and *EBI3* across distinct immune cell subsets. **(G)** Dot plot showing the five-gene hub signature scores across individual immune populations. The identified immune cell subsets included: 1. Germinal center-like B cells; 2. Antibody-secreting cells; 3. Type 1 helper T cells; 4. Activated CD4^+^ T cells; 5. IgA plasma cells; 6. Activated CD8^+^ T cells; 7. Activated B cells; 8. Immature B cells; 9. IgG plasma cells; 10. Macrophages; 11. NK cells; 12. Regulatory B cells; 13. Monocytes; and 14. Plasmacytoid dendritic cells.

Cell-type-specific expression analysis revealed distinct expression patterns of the five hub genes across immune cell populations. *CCR7* was predominantly expressed in activated CD4^+^ T cells and type 1 helper T cells. *CD1D* was highly expressed in macrophages and plasmacytoid dendritic cells. *PTGER2* was predominantly expressed in macrophages and regulatory B cells. *PIK3CG* was broadly expressed in macrophages, monocytes, and activated CD8^+^ T cells. *EBI3* was primarily expressed in antibody-secreting cells and plasma cells (**Figures 7B–F**).

To integrate these findings, a composite hub-gene signature score was calculated across immune cell populations, revealing the highest activity in macrophages, plasmacytoid dendritic cells, and plasma cells, followed by Th1 and activated CD4^+^ T cells (**Figure 7G**). Collectively, these findings demonstrate that the identified hub genes are preferentially expressed in antigen-presenting cells and adaptive immune cell populations, highlighting their close association with the immune landscape of gastric cancer.

### Experimental validation of hub gene expression in clinical EOGC samples

To experimentally validate the bioinformatics-derived hub genes, clinical gastric cancer specimens from EOGC and LOGC patients were examined using qRT-PCR, Western blotting, and IHC. The clinicopathological characteristics of these six samples are summarized in **Supplementary Table 4**. qRT-PCR analysis revealed significantly elevated transcript levels of *CCR7, CD1D, PTGER2, PIK3CG*, and *EBI3* in EOGC tissues relative to LOGC tissues (**Figure 8A**). At the protein level, Western blotting demonstrated a concordant increase in the abundance of these five candidates in EOGC samples (**Figure 8B**). Moreover, IHC staining confirmed stronger protein expression and distribution of the hub genes within EOGC tissues, and semi-quantitative IHC scoring showed consistent results with molecular assays (**Figure 8C**). These findings provide independent clinical evidence supporting the association of the identified hub genes with the EOGC phenotype.

**Figure 8:**
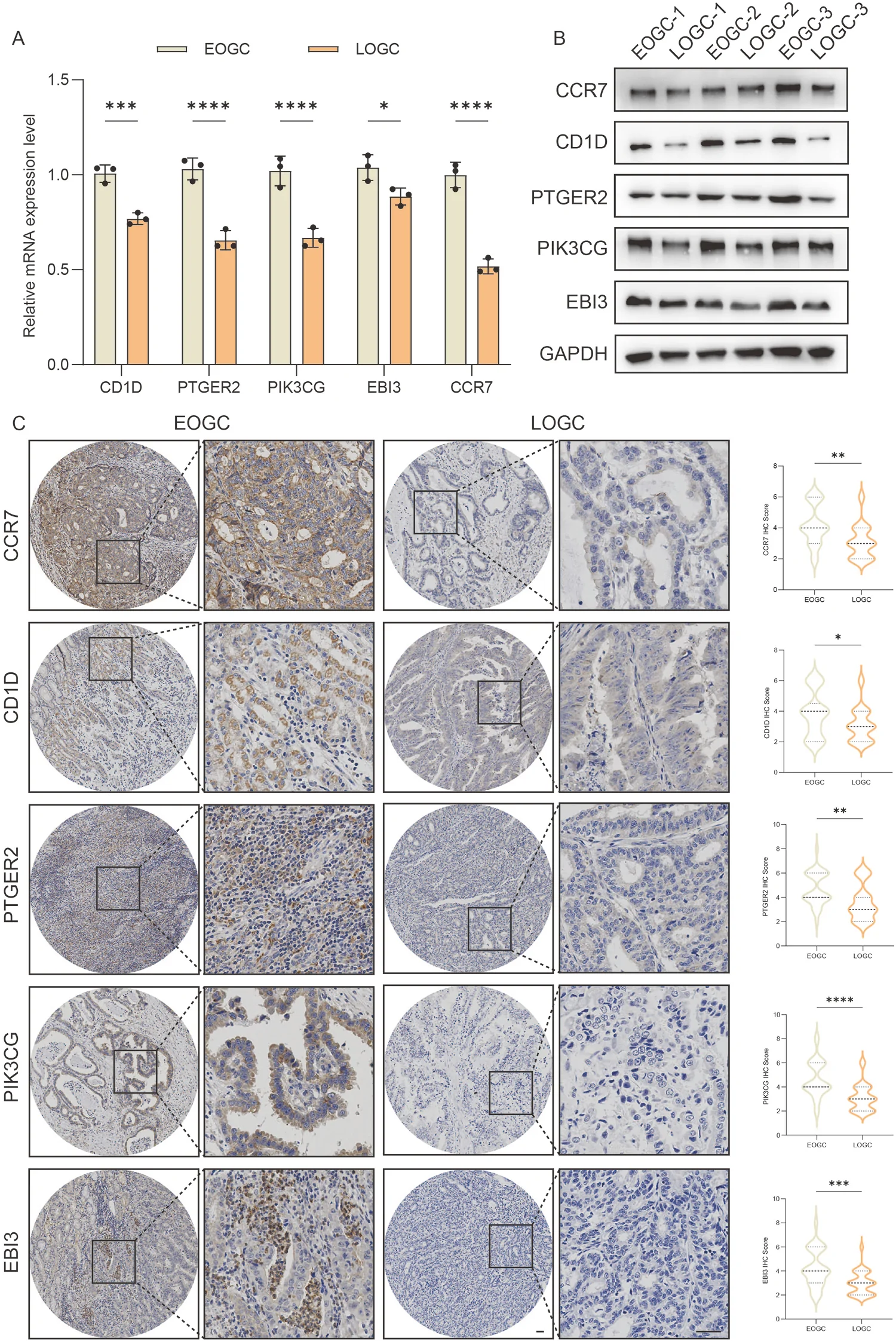
Clinical validation of EOGC-associated hub genes at the mRNA and protein levels. **(A)** Relative mRNA expression levels of *CCR7, CD1D, PTGER2, PIK3CG*, and *EBI3* in EOGC and LOGC tissues assessed by quantitative real-time PCR (qRT-PCR). **(B)** Western blot analysis of protein levels of the five hub genes in EOGC and LOGC tissue specimens. **(C)** Representative immunohistochemical images and quantification of *CCR7, CD1D, PTGER2, PIK3CG*, and *EBI3* protein expression in EOGC and LOGC tissues. Scale bars: 50 μm. * p < 0.05; ** p < 0.01; *** p < 0.001;**** p < 0.0001.

### Hub genes promote gastric cancer cell proliferation and migration

To determine whether the identified hub genes have functional relevance in gastric cancer progression, siRNA-mediated loss-of-function experiments were performed in AGS cells targeting *CCR7, CD1D, PTGER2, PIK3CG*, and *EBI3*.

Silencing of each candidate gene significantly impaired the aggressive cellular phenotypes of AGS cells. Functional assays demonstrated that depletion of these genes reduced proliferative activity, as evidenced by decreased CCK-8 viability signals and diminished colony formation ability (**Figures 9A–C**). Furthermore, suppression of the hub genes markedly inhibited cell migration, as demonstrated by both Transwell assays and wound closure analyses (**Figures 9D–G**).

**Figure 9:**
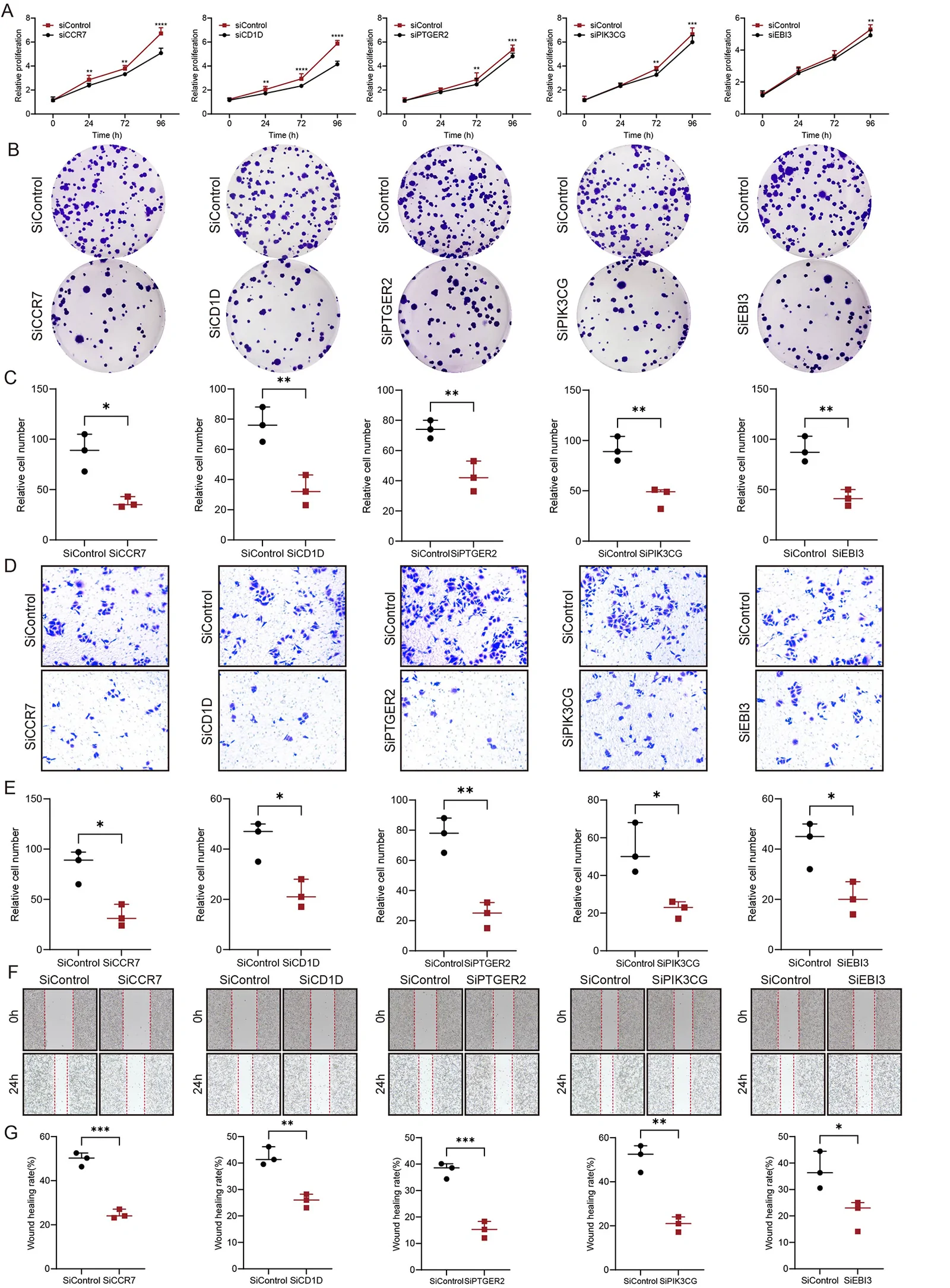
Functional characterization of EOGC-associated hub genes using siRNA-mediated knockdown in gastric cancer cells. **(A)** CCK-8 assays assessing the effects of *CCR7, CD1D, PTGER2, PIK3CG*, and *EBI3* silencing on AGS cell proliferation compared with the siControl group. **(B)** Representative images of colony formation assays after silencing of the five hub genes. **(C)** Quantification of colony numbers in AGS cells following gene silencing. **(D)** Representative images of Transwell migration assays showing reduced migratory ability of AGS cells after hub gene silencing. **(E)** Quantification of migrated cells in Transwell assays. **(F)** Representative images of wound-healing assays evaluating AGS cell migration after hub gene knockdown. **(G)** Quantification of wound closure rates following gene silencing. Statistical significance was defined as follows: * p < 0.05; ** p < 0.01; *** p < 0.001;**** p < 0.0001.

Collectively, these findings demonstrate that *CCR7, CD1D, PTGER2, PIK3CG*, and *EBI3* promote the proliferative and migratory capacities of gastric cancer cells, supporting their potential roles in gastric cancer progression.

## Discussion

EOGC has emerged as a distinct clinical subtype characterized by unique biological features that differ from those of LOGC. However, the molecular basis driving the aggressive phenotype of EOGC remains poorly characterized. In this study, we integrated transcriptomic profiles from multiple independent cohorts and applied a comprehensive analytical framework incorporating WGCNA, machine learning, immune infiltration profiling, single-cell transcriptomics, and experimental validation to elucidate the molecular landscape of EOGC. Our results demonstrate that EOGC exhibits an immune-enriched transcriptional phenotype accompanied by relative suppression of metabolic pathways. Furthermore, we identified five immune-related hub genes that were consistently upregulated in EOGC, validated across independent datasets and clinical specimens, and functionally associated with gastric cancer cell proliferation and migration. Together, these results provide new perspectives on the immunological features of EOGC and identify an EOGC-associated molecular signature that captures transcriptomic characteristics distinguishing EOGC from LOGC.

A major finding of this study is that immune-related activation, rather than metabolic alterations, represents a prominent molecular characteristic of EOGC. Differential expression analysis and functional enrichment consistently demonstrated activation of antigen presentation, cytokine signaling, chemokine signaling, and interferon-related immune pathways, whereas multiple metabolic pathways were relatively suppressed. These observations suggest that EOGC possesses a transcriptional program distinct from that of LOGC and support the concept that age-related gastric cancer subtypes differ not only in clinicopathological characteristics but also in immune biology. Previous transcriptomic studies of EOGC have primarily focused on genomic alterations, hereditary susceptibility, or epithelial tumor-intrinsic pathways (4, 18, 22). By contrast, our findings highlight immune regulation as a central feature of EOGC and provide a complementary perspective on its biological characteristics.

Through integration of WGCNA and two independent machine learning algorithms, we further identified five hub genes that were consistently associated with the EOGC phenotype. Unlike conventional differential expression analysis alone, this multi-step strategy reduced false-positive candidates by incorporating co-expression relationships and independent feature-selection approaches, thereby improving the robustness of hub gene identification. The robustness of this five-gene signature was further supported by validation in an independent cohort, with consistent expression patterns confirmed at both the mRNA and protein levels in clinical specimens. Importantly, the five-gene model should be interpreted as an EOGC-associated molecular signature reflecting transcriptomic differences between EOGC and LOGC, rather than as a stand-alone clinical diagnostic tool.

The pharmacological relevance of the five hub genes also warrants consideration. *CCR7* is a chemokine receptor involved in *CCL19*/*CCL21*-mediated immune-cell trafficking and lymphatic migration, and therapeutic strategies targeting the *CCL19/CCL21/CCR7* axis have been explored in preclinical cancer models, although no clinically established *CCR7*-targeted therapy is currently available (23). *CD1D* is a key antigen-presenting molecule for invariant natural killer T-cell activation, and α-galactosylceramide (KRN7000), a *CD1D*-restricted ligand, has been evaluated in early-phase clinical studies as an immunotherapeutic strategy, although clinical responses have remained limited (24, 25). *PTGER2* encodes the prostaglandin E2 receptor EP2, for which selective antagonists such as PF-04418948 have been developed (26). Importantly, pharmacological or genetic inhibition of EP2 has been reported to suppress gastric cancer growth, induce apoptosis, and enhance sensitivity to chemotherapy, supporting the potential relevance of the PGE2-EP2 axis to gastric cancer therapy (27). *PIK3CG* encodes the PI3Kγ catalytic subunit and represents the most pharmacologically developed target among the five genes. Selective PI3Kγ inhibition by agents such as eganelisib (IPI-549) has entered early-phase clinical studies in patients with advanced solid tumors, supporting the clinical tractability of this pathway (28, 29). *EBI3*, a component of the *IL-27* and *IL-35* cytokine complexes, represents a more context-dependent and less clinically mature therapeutic target. Preclinical studies have shown that *EBI3* blockade can enhance antitumor cytotoxic T-cell responses and suppress tumor growth, suggesting that *EBI3*-associated cytokine signaling may represent a potential therapeutic avenue (30, 31).Taken together, these observations indicate that the five hub genes have different degrees of pharmacological tractability, ranging from experimentally targetable immune and signaling pathways to targets with early clinical-stage pharmacological development. However, the present study does not establish that these genes are directly druggable in EOGC, nor does it demonstrate that their expression levels predict response to specific anticancer agents. Therefore, the identified genes should be considered potential therapeutic candidates and mechanistic entry points for future investigation rather than clinically validated drug targets.

Although these hub genes have diverse biological functions, they converge on immune-related processes. *CCR7* is a key regulator of lymphocyte trafficking and dendritic-cell migration (32), whereas *CD1D* mediates lipid antigen presentation to invariant natural killer T cells (33, 34). *EBI3* participates in cytokine signaling through *IL-27* and *IL-35* complexes (35), *PIK3CG* regulates intracellular immune signaling downstream of multiple immune receptors (36), and *PTGER2* mediates prostaglandin E_2_-dependent inflammatory responses (37). Despite their distinct molecular functions, all five genes demonstrated significant associations with immune-related pathways and immune cell infiltration in our analyses, suggesting that they collectively reflect an immune-active state within EOGC rather than acting as isolated molecular events.

Immune infiltration profiling further reinforced this observation. Compared with LOGC, EOGC displayed enhanced enrichment of diverse adaptive and innate immune populations, including activated B cells, effector memory CD8^+^ T cells, neutrophils, eosinophils, mast cells, and Th1 cells. Moreover, four of the five hub genes showed strong positive correlations with antigen-presenting cells and cytotoxic lymphocytes. Single-cell RNA sequencing analysis further demonstrated that these genes were preferentially expressed in macrophages, dendritic cells, plasma cells, activated CD4^+^ T cells, and other immune cell populations, providing cellular-resolution evidence supporting the bulk transcriptomic findings. Together, these complementary analyses consistently indicate that the identified hub genes are closely associated with the immune landscape of EOGC.

Beyond computational analyses, our study incorporated experimental validation to strengthen the biological relevance of the identified biomarkers. Expression of all five hub genes was consistently elevated in clinical EOGC specimens at both the transcript and protein levels, as confirmed by qRT-PCR, Western blotting, and immunohistochemistry. Furthermore, siRNA-mediated knockdown of each hub gene significantly inhibited gastric cancer cell proliferation and migration, indicating that these genes contribute to malignant cellular phenotypes. Although these functional experiments do not establish the precise molecular mechanisms underlying EOGC progression, they provide independent experimental evidence supporting the biological significance of the identified hub genes.

Several strengths distinguish the present study from previous investigations. First, transcriptomic data from multiple independent cohorts were integrated after rigorous batch-effect correction, improving statistical power and reducing cohort-specific bias. Second, the identification of hub genes was achieved by integrating co-expression network analysis with machine learning algorithms, enabling the prioritization of biologically relevant candidates beyond conventional DEG screening. Third, multiple independent validation strategies, including external datasets, clinical specimens, immune infiltration analysis, single-cell transcriptomics, and functional experiments were incorporated, providing convergent evidence supporting the robustness of our findings.

Several limitations of this study warrant consideration. First, although the machine-learning-based feature-selection procedures, including LASSO regression and random forest analysis, were performed using the training cohort after the 7:3 stratified split, the initial DEG analysis and WGCNA were conducted using the complete integrated discovery cohort before sample splitting. Therefore, information from samples subsequently included in the internal validation cohort may have contributed to the initial identification of candidate genes, representing a potential source of information leakage and limiting the interpretation of the internal validation performance as a fully independent estimate. To further assess the robustness and generalizability of the identified five-gene signature, we incorporated an independent external validation cohort comprising the combined GSE62254 and GSE110875 datasets, which were not used for DEG analysis, WGCNA, feature selection, or model construction. Nevertheless, future studies using a strictly separated discovery and validation framework from the initial DEG and WGCNA steps will be important to provide a more rigorous assessment of the predictive performance of the signature. Second, the retrospective design and reliance on publicly available datasets restricted the availability of detailed clinical information, including treatment history, Helicobacter pylori infection status, molecular subtype, and disease stage, which may have contributed to variations in gene expression patterns and immune profiles (38, 39). Third, although clinical validation confirmed differential expression of the identified hub genes, the sample size remained relatively limited and larger prospective cohorts are required for further validation. Fourth, functional studies were performed in a single gastric cancer cell line, and the molecular mechanisms linking these hub genes to immune regulation and tumor progression remain to be elucidated through additional *in vitro* and *in vivo* experiments. Another limitation is the incomplete availability of clinical annotations across the integrated transcriptomic datasets. Although age, sex, tumor stage, histological subtype, and dataset source were evaluated where information was available, the limited number of samples with complete clinical annotations precluded robust multivariable adjustment or stratified analyses. In particular, the presence of Y-chromosome-related DEGs may partly reflect differences in sex composition between EOGC and LOGC groups. Therefore, these genes were interpreted cautiously rather than as direct evidence of EOGC-specific biological alterations. Future studies involving larger, clinically well-annotated cohorts are warranted to determine whether the identified molecular differences remain independent of sex and other clinical factors.

Future studies integrating prospective clinical cohorts, spatial transcriptomics, multiplex spatial imaging, and mechanistic investigations will further clarify how these hub genes regulate cell-cell communication within the EOGC tumor microenvironment. Moreover, future studies may evaluate whether this molecular signature is associated with treatment response or other clinically relevant outcomes, which could help determine its potential relevance to patient stratification and therapeutic research in EOGC.

In conclusion, our study establishes an integrated molecular and immunological framework for understanding EOGC through the combination of multicohort transcriptomic analysis, machine learning, immune profiling, single-cell transcriptomics, and experimental validation. We demonstrate that EOGC is characterized by an immune-enriched tumor microenvironment and identify five immune-related hub genes that are closely associated with immune signaling, immune cell infiltration, and malignant cellular phenotypes. These findings improve our understanding of EOGC biology and identify an EOGC-associated molecular signature that may facilitate future studies of disease biology, molecular stratification, and treatment-related outcomes.

## Data Availability

Publicly available datasets were analyzed in this study. This data can be found here: The datasets supporting the conclusions of this article are publicly available from The Cancer Genome Atlas (TCGA) database (https://portal.gdc.cancer.gov/) and the Gene Expression Omnibus (GEO) database (https://www.ncbi.nlm.nih.gov/geo/). These publicly available datasets can be accessed by researchers for academic and non commercial purposes. All data analyzed in this study were obtained from publicly accessible repositories.
