# Supplementary material for: Integrated bioinformatics and machine learning identify key hub genes in early-onset gastric cancer
Source: Front Oncol. 2026 Oct 14;16:1951255. doi: 10.3389/fonc.2026.1951255 (PMC13714815; doi:10.3389/fonc.2026.1951255)
Supplement: Supplementary file 2 [file SupplementaryFile4.docx]

**Supplementary Figure 1. (A) Comparison of hub gene expression between EOGC and LOGC samples in the independent external validation cohort. (B) ROC curve analysis assessing the discriminatory performance of the five hub genes in the independent external validation cohort.**

**Supplementary Table 1. Baseline Clinical Characteristics of Patients in the Integrated Discovery Cohort (GSE 84437, GSE 26942 and TCGA-STAD).**

| **Characteristics** | **EOGC** | **LOGC** | **P value** |
| --- | --- | --- | --- |
| n | 82 | 936 |  |
| Age, median (IQR) | 39.5 (35, 43) | 64 (56.75, 71) | < 0.001 |
| Sex, n (%) |  |  | 0.023 |
| Female | 36 (3.5%) | 296 (29.1%) |  |
| Male | 46 (4.5%) | 640 (62.9%) |  |
| Stage, n (%) |  |  | 0.607 |
| 1 | 6 (1.1%) | 93 (16.5%) |  |
| 2 | 6 (1.1%) | 148 (26.2%) |  |
| 3 | 16 (2.8%) | 211 (37.4%) |  |
| 4 | 6 (1.1%) | 78 (13.8%) |  |
| Stage_N, n (%) |  |  | 0.558 |
| 0 | 7 (1.6%) | 73 (16.9%) |  |
| 1 | 24 (5.5%) | 164 (37.9%) |  |
| 2 | 12 (2.8%) | 120 (27.7%) |  |
| 3 | 5 (1.2%) | 28 (6.5%) |  |
| Stage_T, n (%) |  |  | 0.354 |
| I | 0 (0%) | 11 (2.5%) |  |
| II | 4 (0.9%) | 34 (7.9%) |  |
| III | 7 (1.6%) | 85 (19.6%) |  |
| IV | 37 (8.5%) | 255 (58.9%) |  |
| Lauren, n (%) |  |  | 0.955 |
| Intestinal | 15 (8.3%) | 118 (65.6%) |  |
| Diffuse | 5 (2.8%) | 35 (19.4%) |  |
| Mixed | 1 (0.6%) | 6 (3.3%) |  |

**Supplementary Table 2. Baseline Clinical Characteristics of Patients in the Independent External Validation Cohort (GSE 110875 and GSE 62254).**

| **Characteristics** | **LOGC** | **EOGC** | **P value** |
| --- | --- | --- | --- |
| n | 272 | 108 |  |
| Age, median (IQR) | 65 (58, 70) | 39.5 (35.75, 42) | < 0.001 |
| Sex, n (%) |  |  | < 0.001 |
| Male | 189 (49.7%) | 43 (11.3%) |  |
| Female | 83 (21.8%) | 65 (17.1%) |  |
| Stage, n (%) |  |  | 0.196 |
| I | 29 (7.7%) | 5 (1.3%) |  |
| II | 90 (23.8%) | 35 (9.3%) |  |
| III | 86 (22.8%) | 43 (11.4%) |  |
| IV | 65 (17.2%) | 25 (6.6%) |  |
| Stage_N, n (%) |  |  | 0.116 |
| 0 | 35 (11.7%) | 3 (1%) |  |
| 1 | 123 (41%) | 8 (2.7%) |  |
| 2 | 72 (24%) | 8 (2.7%) |  |
| 3 | 42 (14%) | 9 (3%) |  |
| Stage_T, n (%) |  |  | 0.317 |
| 2 | 172 (57.7%) | 14 (4.7%) |  |
| 3 | 79 (26.5%) | 12 (4%) |  |
| 4 | 19 (6.4%) | 2 (0.7%) |  |
| Stage_M, n (%) |  |  | 0.170 |
| 0 | 250 (83.3%) | 23 (7.7%) |  |
| 1 | 22 (7.3%) | 5 (1.7%) |  |
| Lauren, n (%) |  |  | < 0.001 |
| Intestinal | 145 (48.3%) | 5 (1.7%) |  |
| Diffuse | 119 (39.7%) | 23 (7.7%) |  |
| Mixed | 8 (2.7%) | 0 (0%) |  |

**Supplementary Table 3. Performance indices for the five-gene model.**

| **Performance index** | **Training cohort** | **Internal validation cohort** | **External cohort** |
| --- | --- | --- | --- |
| ROC-AUC | 0.909 (95% CI: 0.880-0.938) | 0.859 (95% CI 0.818-0.899) | 0.802 (95% CI 0.758-0.846) |
| Bootstrap AUC | 0.908(95% CI: 0.876-0.921) | 0.852 (95% CI: 0.80020.878) | 0.789 (95% CI 0.743-0.838) |
| Calibration (*P* value) | 0.24 | 0.18 | 0.33 |
| Sensitivity (%) | 0.850 (95% CI: 0.730-0.900) | 0.855 (95% CI 0.729-0.922) | 0.798 (95% CI 0.659-0.855) |
| Specificity (%) | 0.817 (95% CI: 0.725-0.883) | 0.733 (95% CI 0.627-0.807) | 0.720 (95% CI 0.560-0.787) |
| PR-AUC | 0.905 (95% CI: 0.871-0.932) | 0.869 (95% CI 0.822-0.907) | 0.758 (95% CI 0.693-0.820) |
| Balanced accuracy | 0.833 (95% CI: 0.792-0.870) | 0.794 (95% CI 0.749-0.834) | 0.759 (95% CI 0.713-0.803) |

**Supplementary Table 4. Clinicopathological characteristics of gastric cancer patients for experiment validation.**

| **Type** | **Sex** | **Age** | **TNM stage** | **Tumor stage** | **Histological sbtype** | **Adenocarcinoma differentiation** |
| --- | --- | --- | --- | --- | --- | --- |
| LOGC | Male | 54 | T2N1M0 | IIA | Adenocarcinoma | Well differentiated |
| LOGC | Female | 67 | T4aN2M0 | IIIA | Adenocarcinoma | Moderately to poorly differentiated |
| EOGC | Male | 41 | T4aN3aM0 | IIIB | Adenocarcinoma with signet ring cell carcinoma | Poorly differentiated |
| EOGC | Male | 36 | T3N2M0 | IIIA | Adenocarcinoma | Poorly differentiated |
| EOGC | Male | 43 | T3N1M0 | IIB | Adenocarcinoma with signet ring cell carcinoma | Poorly differentiated |
| LOGC | Female | 68 | T2N1M0 | IIA | Adenocarcinoma | Moderately differentiated |

**Supplementary Table 5. Sequences for siRNAs and shRNAs**

| **Name** | **Sequences (5′ to 3′)** |
| --- | --- |
| SiCCR7 | GGAUCUUGCUACCUACAAATT |
| SiCD1D | GCUCUUCAUCUACAGCAUUTT |
| SiPTGER2 | GCAGAUUGCUCUUCAUCAATT |
| SiPIK3CG | GGACCUUCAUCAAGAUCAATT |
| SiEBI3 | GGAUCUUCCUGGAAUACAATT |

**Supplementary Table 6. Primers for qPCR**

| **Gene** | **Sequences (5′ to 3′)** | **Application** |
| --- | --- | --- |
| CCR7 | Forward: TCTGGACCTGCTGCTCATCT | qPCR |
|  | Reverse: AGGTTGAGCTGCTTGGTGTT |  |
| CD1D | Forward: TGGAGGCTCTGTTCTTCCTG | qPCR |
|  | Reverse: CAGGTTGTTGGTGAGGTTGG |  |
| PTGER2 | Forward: GCTGCTCATCTACTTCGCCA | qPCR |
|  | Reverse: AGGTAGCGGTAGGTGATGGT |  |
| PIK3CG | Forward: CCTGACCTTCATCGAGGACA | qPCR |
|  | Reverse: GTCTTCAGGATGGCCTTGGT |  |
| EBI3 | Forward: TGCTGCTGATGCTGTGTTCT | qPCR |
|  | Reverse: TGGTTGTAGTCGCTGGTGAT |  |
| GAPDH | Forward: GGAGCGAGATCCCTCCAAAAT | qPCR |
|  | Reverse: GGCTGTTGTCATACTTCTCATGG |  |

**Supplementary Table 7. Antibodies**

| **Antigen** | **Manufacturer** | **Catalog Number** | **Application** |
| --- | --- | --- | --- |
| CCR7 | servicebio | GB11502 | 1:1000 for WB |
|  |  |  | 1:300 for IHC |
| CD1D | Proteintech | 66257-1-Ig | 1:50 for WB |
|  |  |  | 1:200 for IHC |
| PTGER2 | servicebio | GB111814 | 1:1000 for WB; 1:500 for IHC |
| PIK3CG | Proteintech | 31229-1-AP | 1:1000 for WB 1:500 for IHC |
| EBI3 | Proteintech | 12371-1-AP | 1:1000 for WB |
| β-actin | Proteintech | 66009-1-Ig | 1:20000 for WB |
| anti-mouse IgG HRP-linked Ab | Cell Signaling Technology | 7076 | 1:1000 for WB |
| anti-rabbit IgG HRP-linked Ab | Cell Signaling Technology | 7074 | 1:1000 for WB |

**Supplementary Table 8. Abbreviation**

| **Abbreviation** | **Full Name** |
| --- | --- |
| EOGC | Early-onset gastric cancer |
| LOGC | Late-onset gastric cancer |
| TCGA | The Cancer Genome Atlas |
| GEO | Gene Expression Omnibus |
| TCGA-STAD | TCGA stomach adenocarcinoma |
| sva | Surrogate Variable Analysis |
| WGCNA | Weighted Gene Co-expression Network Analysis |
| GS | Gene Significance |
| MM | Module Membership |
| Reactome | Reactome Pathway Database |
| LASSO | Least Absolute Shrinkage and Selection Operator |
| ROC | Receiver Operating Characteristic |
| AUC | Area Under the Curve |
| GSEA | Gene Set Enrichment Analysis |
| ssGSEA | Single-sample Gene Set Enrichment Analysis |
| SVR | Support Vector Regression |
| TRIzol | TRIzol Reagent |
| cDNA | Complementary DNA |
| qRT-PCR | Quantitative Real-time PCR |
| GAPDH | Glyceraldehyde 3-phosphate dehydrogenase |
| RIPA | Radioimmunoprecipitation Assay Buffer |
| BCA | Bicinchoninic Acid Assay |
| SDS-PAGE | Sodium Dodecyl Sulfate Polyacrylamide Gel Electrophoresis |
| PVDF | Polyvinylidene Fluoride Membrane |
| ECL | Enhanced Chemiluminescence |
| IHC | Immunohistochemistry |
| DAB | 3,3′-Diaminobenzidine |
| CCK-8 | Cell Counting Kit-8 |
| FBS | Fetal Bovine Serum |
