# Supplementary material for: Integrated bioinformatics and machine learning identify key hub genes in early-onset gastric cancer
Source: Front Oncol. 2026 Oct 14;16:1951255. doi: 10.3389/fonc.2026.1951255 (PMC13714815; doi:10.3389/fonc.2026.1951255)
Supplement: Supplementary file 3 [file SupplementaryFile1.docx]

| CCR7 |  |  |
| --- | --- | --- |
| PIK3CG |  |  |
| EBI3 |  |  |
| PTGER2 |  |  |
| CD1D |  |  |
| GAPDH |  |  |
